# Use of autologous bone graft in anterior cervical decompression: morbidity & quality of life analysis

**DOI:** 10.1186/1471-2474-10-158

**Published:** 2009-12-16

**Authors:** Helen M Heneghan, John P McCabe

**Affiliations:** 1 Department of Surgery, Clinical Science Institute, National University of Ireland Galway, Galway, Ireland; 2 Department of Trauma and Orthopaedics, Galway University Hospital, Galway, Ireland; 3 Department of Orthopaedic Surgery, Merlin Park Hospital, Galway, Ireland

## Abstract

**Background:**

Autologous iliac crest graft has long been the gold standard graft material used in cervical fusion. However its harvest has significant associated morbidity, including protracted postoperative pain scores at the harvest site. Thus its continued practice warrants scrutiny, particularly now that alternatives are available. Our aims were to assess incidence and nature of complications associated with iliac crest harvest when performed in the setting of Anterior Cervical Decompression (ACD). Also, to perform a comparative analysis of patient satisfaction and quality of life scores after ACD surgeries, when performed with and without iliac graft harvest.

**Methods:**

All patients who underwent consecutive ACD procedures, with and without the use of autologous iliac crest graft, over a 48 month period were included (n = 53). Patients were assessed clinically at a minimum of 12 months postoperatively and administered 2 validated quality of life questionnaires: the SF-36 and Cervical Spine Outcomes Questionnaires (Response rate 96%). Primary composite endpoints included incidence of bone graft donor site morbidity, pain scores, operative duration, and quality of life scores.

**Results:**

Patients who underwent iliac graft harvest experienced significant peri-operative donor site specific morbidity, including a high incidence of pain at the iliac crest (90%), iliac wound infection (7%), a jejunal perforation, and longer operative duration (285 minutes vs. 238 minutes, p = 0.026). Longer term follow-up demonstrated protracted postoperative pain at the harvest site and significantly lower mental health scores on both quality of life instruments, for those patients who underwent autologous graft harvest

**Conclusion:**

ACD with iliac crest graft harvest is associated with significant iliac crest donor site morbidity and lower quality of life at greater than 12 months post operatively. This is now avoidable by using alternatives to autologous bone without compromising clinical or technical outcome.

## Background

Historically, autologous bone graft harvested from the iliac crest has been the graft material of choice utilised in spinal fusion surgery. Favouring it's use are its' osteogenic, osteoinductive and osteoconductive properties in addition to being histocompatible and completely osteointegrative [1]. However, it remains a technique with significant morbidity and with the advent of newer, viable alternatives such as structural allografts and artificial disc replacements, it's continued practice warrants further scrutiny. The most significant disadvantage of using autogenous bone graft in spinal fusion surgery is the associated donor site morbidity, with reported incidence of 10-50% in the literature [2-6]. Numerous reports in the published literature site major complications associated with this technique including neurovascular injury, deep wound infection, haematoma, peritoneal perforation and ureteral injury. Chronic complications include donor site pain, herniation, meralgia paraesthetica and avulsion fractures of the anterior superior iliac spine. It is widely acknowledged that cervical fusion prrovides excellent clinical results, however it is not without adverse sequelae, including increased biomechanical stress at levels adjacent to the fused segment. There is currently limited data available juxtaposing the outcomes from cervical discectomy and fusion with contemporary disc arthroplasty procedures. Long term follow-up for disc replacement remains incomplete, but encouragingly short-term clinical results are comparable to spinal fusion procedures [7,8].

Whilst there are infrequent reports chroniciling the incidence and range of complications of iliac crest harvest [2-6], no available data have directly compared Anterior Cervical Decompression and Fusion (ACDF) using iliac bone graft with newer synthetic alternatives, in relation to addressing donor site morbidity and in particular no study has previously assessed the quality of life or satisfaction of patients after iliac bone autograft harvest in this setting. Silber and colleagues concluded from their assessmnet of donor site morbidity after iliac graft harvest that this procedure warrants extreme caution, and that alternative sources of graft material must be considered given the potential adverse clinical complications [6].

The purpose of this study is to assess the incidence and nature of complications associated with autologous iliac crest graft harvest in our unit, where performed in the setting of ACDF. Aditionally we wished to assess patient satisfaction after ACD procedures, and compare quality of life outcomes of patients who underwent autologous graft harvest with patients who did not.

## Methods

### Study design

This study was a retrospective assessment of consecutive patients (N = 53) who underwent primary Anterior Cervical Decompression (ACD) by a single surgeon in a tertiary referral spinal unit, over a 46 month period from March 2004 to December 2007. Ethical approval for this study was sought and granted by our local ethics committee (Galway University Hospitals Clinical Research Ethics Committee).

### Inclusion and exclusion criteria

We identified all patients who had primary ACD over one to three levels, for all cervical spine pathology resulting in cervical myelopathy or radiculopathy. Diagnoses were formulated by a combination of clinical history, physical examination, plain radiograph of the cervical spine, and more complex imaging modalities such as magnetic resonance imaging and/or computed tomography. The resultant diagnoses included degenerative cervical spine disease, trauma and myeloma of the cervical spine (Table 1). Exclusion criteria (n = 5) included death at follow-up (n = 1), patients who had previous cervical spine surgery (n = 3) and refusal to participate in the study (n = 1).

**Table 1:** Baseline patient characteristics

	No Bone Graft (n = 18)	Bone Graft (n = 29)	*p value*
Mean age	50 years (37-62)	53 years (36-66)	0.196
Ex/Current smoker	50%	58%	*0.109*
Mean Follow-up	15 months	25 months	
(Range)	(12-43)	(12-46)	*0.016*
Operative level			
1	56%	42%	*0.718*
2	39%	48%	
3	5%	10%	
Aetiology			
Degenerative	83%	83%	
Trauma	17%	14%	*0.723*
Other	0%	3%	

### Study participants and follow-up

**Figure 1:**
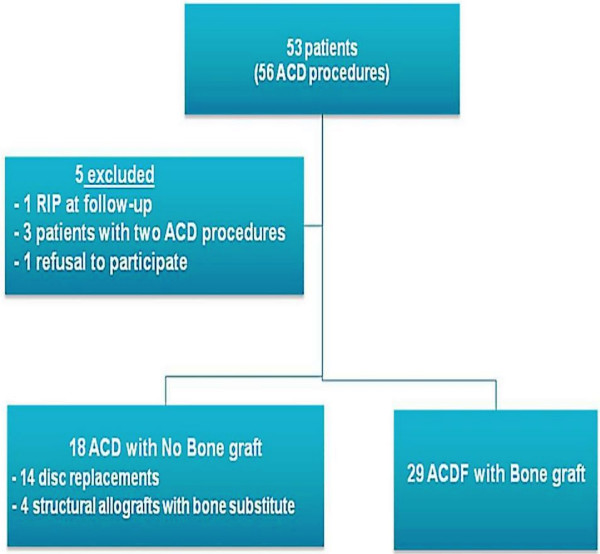
**53 patients underwent ACD over the study period (34 months)**. After applying our exclusion criteria 47 patients were included in the study; 29 of whom underwent ACD with use of autologous iliac crest bone graft and 18 underwent ACD without use of bone graft.

Of the 47 patients who met the inclusion criteria, 29 underwent ACD and fusion using iliac crest bone graft and 18 underwent ACD with either structural allograft (cage and synthetic bone substitute, n = 4) or disc replacement (n = 14) (Fig 1). The decision to utilise autologous bone or its alternatives was established by the operating surgeon preoperatively and each patient gave informed written consent prior to surgery. Both groups, those having bone graft harvesting or synthetic substitutes, were comparable in terms of demographics, disease aetiology and anatomical site of surgery. Both groups also completed a minimum of 12 months follow-up, although mean follow-up was slightly longer overall for the cohort who underwent iliac crest grafting (Table 1). As disc arthroplasty and synthetic bone substitutes for use in structural allografts are relatively new devices in spinal practice their routine use has only evolved in our unit over the last 2.5 years. Prior to their introduction, iliac crest graft was routinely harvested to augment osseous union in ACDF procedures. Consequently, mean follow-up for the group of patients who underwent ACDF with bone graft alternatives is significantly shorter when compared to the group who had bone graft harvested (15 months (Range 12-43) vs 25 months (Range 12-46), p = 0.016).

### Surgical and postoperative protocols

All patients had a standard operative approach to the cervical spine, through a left sided collar incision, with the operating surgeon using Loupes and headlight initially, with microscopic visualisation at the cervical disc level. The Smith-Robinson technique was used for ACDF, which incuded complete discectomy and burring down of the uncinate processes in those patients with foraminal stenoses. For patients undergoing ACDF with harvesting of bone graft, an inter-vertebral cage (*Cornerstone^® ^cage, Medtronic Sofamor Danek, Memphis, TN*) was placed in the space created by the discectomy, and was filled with bone harvested from the left anterior iliac crest in all cases. A corticocancellous graft (30-50 cc volume) was harvested through an open, lateral approach to the anterior ilium. Those undergoing ACDF with structural allograft again had an inter--vertebral cage (*Cornerstone^® ^cage, Medtronic Sofamor Danek, Memphis, TN*) placed in the space created by the discectomy and the cage was filled with synthetic bone substitute, namely a tricalcium phospahte osteoconductive material (Mastergraft^® ^Medtronic Sofamor Danek, Memphis, TN). For those patients having disc arthroplasty, the Prestige^® ^disc was used.

All patients had a deep suction drain placed at the operative site, and received prophylactic intravenous antibiotics at induction of anaesthesia. Antibiotics were continued for 24 hours post-operatively. All patients were monitored for 24 hours in a high dependency unit prior to transfer to a dedicated orthopaedic ward.

### Follow-up & assessment of outcomes

Following discharge after ACD procedures, all patients were reviewed in the out-patient clinic at weeks 2,6,12 and 24, and at six monthly intervals thereafter. In July 2008 each patient in this study was again reviewed clinically and consent obtained to proceed with the administration of two questionnaires. A single clinician administered these questionnaires to each of the study participants; the Cervical Spine Outcome Questionnaire, derived and validated in Johns Hopkins Spinal Unit in 2002 [9] and the widely used and validated SF-36 quality of life questionnaire [10]. Permission to use these questionaires was sought from, and granted by, the authors or regulating bodies governing their circulation. Formal analysis of questionnaire responses was conducted using questionnaire-specific scoring systems provided by their respective authors.

The SF-36 survey measures eight health concepts; physical functioning, social functioning, role limitations due to physical health problems, role limitations due to emotional problems, general mental health, general health perceptions, bodily pain and vitality. The commonest method of reporting these results is by grouping the 8 concepts into two aggregate summary measures - thereby giving a physical health score and a mental health score. Current recommendations involve a 'norm based' scoring system which standardizes each of the 8 scales, allows for easier interpretation, and makes comparisons of the scales possible. When these two summary scores are norm-based, the resulting score may range from 0 to 100. Any time a score is greater than 50 it implies that this outcome is better than the general population average for that measure. Similarly, any time the summary scores are less than 50, the outcome is poorer than the general population average for that measure.

The Cervical Spine Outcomes Questionnaire is a comprehensive disease specific instrument which evaluates the outcomes of treatments for neck and/or arm pain resulting from cervical spine pathology. It tabulates results in the form of six composite measures including two pain severity measures (one for neck pain and one for arm pain), a functional disability measure, a psychological distress measure, a physical symptom measure and a healthcare utilisation measure which includes use of narcotic analgaesics and psychoactive drugs. A specific formula is required to calculate the six scores from the questionnaire responses (available from the author, BenDebba M), and the scores all range from 0-100 with a higher score indicating greater overall severity of pain, functional disability, psychological distress, physical symptoms and healthcare utilisation respectively.

Operative and clinical notes were also analyzed for total operative time, estimated blood loss, length of hospital stay, complications and postoperative morbidity, and subsequent readmission to hospital. Primary endpoints of this study were the incidence of bone graft donor site morbidity, pain scores - which were assessed using a 5 point adjective rating scale, cervical spine specific symptom outcomes, and overall quality of life scores. These endpoints were measured at a minimum of 12 months (Range: 12-46 months; Mean: 25 and 15 months in the groups who did and did not undergo bone graft harvesting respectively) following the ACD procedures.

### Statistical analysis

Data were analysed using the software package SPSS 15.0 for Windows. Normality of the data was checked using the Kolmogorov-Smirnov test and statistical comparisons were made between the two ACD study groups (those who had bone graft harvested and those who did not) using standard descriptive analyses, and parametric tests. Fisher's exact test (two-sided) was used to analyse categorical variables whilst Student's t-tests were used to compare means between the two groups where appropriate. Probability values of less than 0.05 were assumed to represent statistical significance.

## Results

Over the 46 month duration of this study, 53 patients underwent 57 Anterior Cervical decompression procedures by a single spinal surgeon in a tertiary referral orthopaedic unit. Based on inclusion criteria, 47 patients were included for analysis. This included 29 who underwent ACDF with bone graft harvested from the left anterior iliac crest, and 18 who underwent ACD without bone graft, using the alteratives of disc arthroplasty or synthetic bone substitute (Fig 1). The groups were comparable in terms of baseline demographics, operative cervical spine level, and aetiology of the cervical spine disease (Table 1). There was no significant difference between the groups with regard to their preoperative presentations. Similar proportions presented to the spinal unit with cervical radiculopathy and myelopathy; also the presence of preoperative neurological deficit did not differ significantly between the two groups (28% in ACDF & bone graft group vs 55% in ACDF without bone autologous graft, p = 0.071, *Fisher's exact test*).

There was no difference between the two groups with respect to length of hospital stay or intraoperaitve blood loss (Table 2). As expected, patients who underwent iliac crest graft harvest at ACDF had a significantly longer operative duration for (285 minutes vs 238 minutes, p = 0.026, *t-test*).

**Table 2:** Operative details

	No Bone Graft (n = 18)	Bone Graft (n = 29)	*p value*
Mean length of stay	7.0 days (3-16)	7.04 days (4-18)	0.732
Intra-op blood loss	< 100 ml	< 100 ml	0.576
Operative duration	238 min	285 min	0.026

Both groups had similar incidences of postoperative complications overall - 17% incidence in both groups (n = 3 in the cohort who underwent iliac graft harvest and n = 5 in the cohort who had ACD without autologous bone graft). Of the five patients in the autologous bone graft group who did experience a complication, three of these were related directly to the graft harvest itself (Table 3).

**Table 3:** Post-operative morbidity other than pain

	No Bone Graft (n = 18)	Bone Graft (n = 29)	*p value*
Overall complications	17% (n = 3)	17% (n = 5)	*0.959*
Neck wound haematoma & infection	5.6% (n = 1)	6.9% (n = 2)	
Horner's syndrome	5.6% (n = 1)	0	
LRTI	5.6% (n = 1)	0	
Iliac crest wound infection	n/a	6.9% (n = 2)	
Bowel perforation	n/a	3.45% (n = 1)	

**Figure 2:**
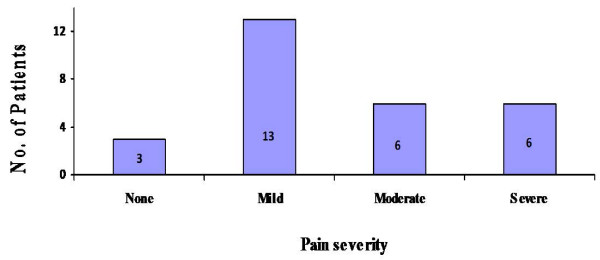
**Subjective postoperative pain scores at the donor site following iliac crest harvest, as measured using a 5 point adjective rating scale**.

Regarding post-operative pain scores associated with the iliac crest graft harvest, 90% (n = 25) of our study participants who underwent autologous bone graft harvest, experienced pain at the graft site for more than one month postoperatively. This was of greater than 12 weeks duration in 38% (n = 11) and the mean duration of pain overall was 13.3 weeks. Twelve patients (43%) reported the severity of this pain as moderate or severe for its total duration (Fig 2).

There was a 7% (n = 2) incidence of iliac crest wound infection in our study group. The other significant complication specific to the iliac graft harvest involved a 43 year old female who sustained a bowel perforation from a small spiculated fragment of ilium. She had a background history of laparotomy some years previously, and when she developed peritonitis on post-operative day three, she underwent emergency exploratory laparotomy. This revealed a jejunal perforation and she proceeded to undergo limited jejunal resection and primary anastomosis. She had an uneventful post-operative course and remains well at follow-up with no adverse sequelae.

The response rate to our questionnaire-based assessments of patients' quality of life, cervical spine outcomes, and overall satisfaction with their procedure, was 96%. The SF-36 results (Table 4) are given as two aggregate summary measures - a physical health score and mental health score. We found no difference in mean physical health scores between patients who had iliac crest graft harvested for ACDF, and those having synthetic bone substitutes or artificial disc replacement (43.3 vs 42.1 respectively, p = 0.810). However, we did find significantly lower mental health scores with the SF-36 questionnaire for the group who had bone graft harvested (54.11 vs 58.4, p = 0.025), at a minimum of 1 year post-operatively.

**Table 4:** SF-36 Survey mean aggregate summary scores

	No Bone Graft (n = 18)	Bone Graft (n = 29)	*p value*
Physical score	42.09	43.32	0.810
Mental score	58.4	54.11	*0.025*

With regard to Cervical Spine Outcomes Questionnaire results there was a significantly higher mean level of psychological distress amongst the patients who had bone graft harvested compared to patients who did not undergo harvesting of iliac crest graft (38.37 vs 19.14, p = 0.024), at a minimum of 1 year post-operatively. Both groups of patients reported similar degrees of current residual neck/arm pain, functional ability, and healthcare utilisation as measured by this disease specific instrument (Table 5).

**Table 5:** Cervical Spine Outcomes Questionnaire scores

	No Bone Graft (n = 18)	Bone Graft (n = 29)	*p value*
Pain severity, neck	10.37	17.01	*0.139*
Pain severity, arm	7.22	6.44	*0.628*
Functional disability	18.78	21.01	*0.877*
Psychological distress	19.14	38.37	*0.024*
Physical symptoms other than neck/arm pain	12.96	18.07	*0.344*
Healthcare utilisation	24.44	22.07	*0.415*

## Discussion

This study is the first to compare Anterior Cervical Discectomy (ACD) performed with iliac crest bone graft versus structural allograft or artificial discs, with respect to clinical outcomes, complications, and most salient in this study - patient's quality of life and overall satisfaction with their procedure. We found that patients who underwent ACD performed with alternatives to autologous bone, such as synthetic bone substiutes or disc replacements, had similar outcomes in terms of relief of neck or arm pain and improvements in functional ability, when compared to patients who had the traditional procedure using iliac crest graft. Additionally these patients had superior quality of life after ≥ 1 year postoperatively compared to patients who underwent bone graft harvest at the time of ACD surgery.

Prior to the conception of synthetic bone substitutes and artificial cervical discs in recent years, it was routine practice to harvest autologous bone graft for use in ACD and fusion procedures, in order to augment osseous union. In fact Klapp is believed to be the first to describe the method of harvesting bone grafts from the iliac crest in 1917 [11]. Since then the techniques employed to harvest bone from this site have progressed from the historical 'mallet and chisel' technique to use of acetabular reamers or small curettes to extract cancellous bone from between the inner and outer tables of the crest. Injection of local anaesthetic (eg. Marcain 0.5%) into the graft harvest site is a commonly employed technique currently, to try to minimize post-operative pain at the donor site [12].

Although autologous bone graft used in the setting of ACD and fusion offers several advantages over alternative graft materials, its harvest has a significant associated morbidity. Documented donor-site complications include nerve, arterial, or urethral injury; chronic donor-site pain; cosmetic deformity; herniation of abdominal contents; sacroiliac joint instability; pelvic fractures; gait disturbances; hematoma; infection; peritoneal perforation; and hip subluxation [2-6,13]. We reported an incidence of donor site morbidity, other than pain, of 17% which is consistent with published literature. Sixty percent of this morbidity was directly consequent to the graft harvest (3 of 5 patients who experienced a complication), and included wound infections at the harvest site, and a most unfortunate incidence of bowel perforation in a female who had undergone a previous laparotomy predisposimg her to adhesion formation and thus abnormal intraperitoneal anatomy. Data on donor site pain varies greatly in the literature and has been reported to persist for over 3 months in 2.8% to 39% of patients. We report similarly that 38% of patients in our group experienced moderate or severe pain persisting for over 3 months.

Historically, the iliac crest was donor site of choice due to the volume of bone available, and the ease of access for bone harvesting. Though some studies suggest the rate of complications is higher for anterior versus posterior iliac crest harvesting; for ACD procedures it is preferable to use anterior iliac crest as the patient is in the supine position [14,15]. The anterior approach was used for all 29 patients who had bone graft harvested in our series, and the volume of graft harvsested ranged between 30-50 cc.

In our unit, we experienced one very significant complication, namely a jejunal perforation, associated with the harvest of anterior iliac crest graft in 2005. This rare complication was most likely contributed to by the patients numerous previous laparotomies and extensive adhesion fromation. However we believe this occurance provides further stimulus to change from harvesting autologous bone graft, to the newer synthetic alternatives, for use in ACD surgery. Such was the magnitude of this particular morbidity that we feel it is incumbent upon us to highlight it and raise awareness amongst spinal and general orthopaedic surgeons of the potential for such an event when harvesting iliac crest graft. Due to the subsequent good experience in our unit with bone substitute materials and disc arthroplasty over the last three years, in addition to recent publications supporting their use these alternatives to solid fusion have largely supplanted the use of autologous bone graft in ACD surgery [16,17]. Despite a relatively short follow-up for patients having disc arthroplasty or synthetic bone used for ACD, we have witnessed similar outcomes to date with respect to residual postoperative neck pain and disability when compared to the group undergoing traditional iliac crest bone harvest. Further evidence to support the use of alternative materials to autologous bone for ACD surgery is our finding that patients who underwent ACD with iliac crest bone graft had a significantly longer operative duration (285 min vs 238 min, p = 0.026). It is a well established finding that longer anaesthetic duration is associated with significantly higher rates of postoperative morbidiity[18,19]. Whilst we found no significant difference between the two groups with regard to the incidence of postoperative morbidity, despite the fact that the cohort who underwent bone graft harvest endured a longer operative duration, we acknowlegde that our study numbers are currently small, and we believe that the longer anaesthetic duration is nevertheless another disincentive to harvesting autologous bone graft during cervical spine surgery.

With regard to patients' quality of life and overall satisfaction after their cervical decompression procedure, we found that pateints who underwent ACD and harvesting of iliac crest bone graft reported significantly poorer mental health scores and greater levels of psychological distress even as far as one year after the procedure, on quality of life assessment postoperatively. Both questionnaires, one of which was cervical spine specific, gave concordant results with regard to patients psychological outcome, and similarly these quality of life assessment instruments showed that patients don't experience significantly different physical or functional outcomes whether the previous gold standard iliac bone autograft is used or newer alternatives, in the setting of ACDF. Interestingly, many of the patients who scored poorly on psychological outcomes in these questionnaires commented that they attributed their postoperative distress and anxiety to the discomfort and pain they experienced as a result of the graft harvest.

We acknlowledge the limitations that our study numbers are small, our quality of life analysis is retrospective, and that our results regarding morbidity associated with iliac graft harvest are in keeping with previously published data. However with modern health care systems currently acknolwedging the importance of patient satisfaction and quality of life as valid endpoints, then the results from our disease specific quality of life assessments are noteworthy. We also postulate that as our numbers increase, the benefits of disc arthroplasty and bone substitutes will become even more pronounced. However we do believe that a prospective, randomized evaluation of autologous bone graft versus synthetic graft material in ACDF procedures is urgerntly needed to definitively and conclusively address this issue.

Further support for the use of alternatives to autologous bone in the setting of ACD and fusion lies in recent data which suggests that it is more cost effective to use synthetic bone substitutes or allografts compared to ACD and fusion with autograft [20]. Additionally, because these modern treatment options allow near normal range of neck motion postoperatively, in comparison to the restricted range of motion following cervical fusion, this may obviate the requirement for subsequent procedures which have historically been often necessary following solid fusion. Whilst there is no convincing evidence from clinical studies that adjacent segment disease is the direct recult of fusion surgery, studies report between 25 and 92 per cent of patients with cervical spine fusion develop adjacent level disease [21]. However it is thought that whilst increased stress at levels adjacent to a fused segment may be a factor in development of this problem, it is more likely to be multifactorial. In order to truly assess whether cervical fusion, with its associated increase in stress at adjacent levels, is in isolation responsible for clinically significant disease, we must await results of long term studies assessing the occurrence of adjacent level disease in patients who have undergone disc replacement where range of cervical motion is maintained.

## Conclusions

ACD and fusion with bone graft harvested from the iliac crest is associated with significant patient morbidity related specifically to the graft harvest. Use of this technique has been challenged and largely supplanted by disc arthroplasty or the use of synthetic bone substitute, without compromising clinical outcome. Patients' psychological outcome and overall quality of life, at one year or greater postoperatively, is superior after ACD with use of disc arthropolasty or synthetic bone, when compared to ACD and fusion with autologous iliac crest bone graft.

## Competing interests

We, the authors have no competing interests and have no acknowledgements to include.

## Authors' contributions

HMH was primarily responsible for literature search, collation of data, statistical analyses, drafting, and submission and the manuscript. JPM conceived of the study, participated in its design, directed clinical & operative management of the patients, and reviewed the manuscript. All authors read and approved the final manuscript.

## Authors' information

HMH is a surgical research fellow. JPM is a Consultant Spinal Surgeon at Galway University Hospital & at Merlin Park hospital, an affiliated university teaching hospital.

## Pre-publication history

The pre-publication history for this paper can be accessed here:

http://www.biomedcentral.com/1471-2474/10/158/prepub
